# Comparative Assessment of Acute Pulmonary Effects Induced by Heat-Not-Burn Tobacco Aerosol Inhalation in a Murine Model

**DOI:** 10.3390/ijms26031135

**Published:** 2025-01-28

**Authors:** Beong Ki Kim, Won Jin Yang, Ye Seul Seong, Yong Jun Choi, Hye Jung Park, Min Kwang Byun, Yoon Soo Chang, Jae Hwa Cho, Chi Young Kim

**Affiliations:** 1 Division of Pulmonary and Critical Care Medicine, Department of Internal Medicine, Dongguk University College of Medicine, Dongguk University Ilsan Hospital, Goyang 10326, Republic of Korea; bkklung@dumc.or.kr; 2 Department of Internal Medicine, Yonsei University College of Medicine, Seoul 03722, Republic of Korea; octa821@yuhs.ac (W.J.Y.); cyj0717@yuhs.ac (Y.J.C.); craft7820@yuhs.ac (H.J.P.); littmann@yuhs.ac (M.K.B.); yschang@yuhs.ac (Y.S.C.); jhcho66@yuhs.ac (J.H.C.); 3 Division of Pulmonology, Department of Internal Medicine, Yonsei University College of Medicine, Gangnam Severance Hospital, Seoul 06273, Republic of Korea; xochipilli@yuhs.ac; 4 Division of Pulmonary, Allergy, and Critical Care Medicine, Department of Internal Medicine, Korea University College of Medicine, Korea University Ansan Hospital, Ansan 15355, Republic of Korea

**Keywords:** electronic nicotine delivery systems, lung injury, model animal, smoking, toxicity test

## Abstract

Tobacco smoking remains a major global health concern, causing preventable deaths and economic strain. Although new tobacco products such as heat-not-burn (HnB) are safer alternatives to traditional cigarettes, research on their associated risks remains limited. This study aimed to investigate the effects of HnB smoke exposure on the lungs compared to those of traditional cigarettes and the combined use of HnB and cigarettes using experiments with a mouse model. We quantitatively analyzed changes in the levels of 92 blood plasma proteins using the proximity extension assay method and observed significant changes in their levels in mice exposed to different smoke conditions; specifically, the levels of certain proteins, including Ccl20, Cxcl1, and Pdgfb, increased in the HnB smoke-exposed group, suggesting activation of nicotine pathways. Comparative analysis with traditional cigarette smoke-exposed mice further highlighted similarities and differences in their protein expression profiles. This study contributes to an improved understanding of the biological mechanisms underlying the harmful effects of alternative nicotine delivery systems and identifies potential biomarkers associated with the harmful effects of HnB smoke exposure. However, the precise impact of nicotine on the immune system may be influenced by various factors, necessitating further research.

## 1. Introduction

Tobacco smoking remains a major global public health issue, contributing substantially to preventable deaths worldwide. Tobacco use involves a complex mixture of over 5000 toxic and carcinogenic substances [1] and is a well-established risk factor associated with cardiovascular diseases, chronic obstructive pulmonary disease, and various cancers [2,3,4,5]. Despite extensive public health efforts, 1.14 billion people smoked in 2019, resulting in 7.69 million deaths and 200 million disability-adjusted life-years globally [6]. This situation creates substantial health and economic challenges, leading to considerable direct healthcare expenses [7].

Various tobacco products have emerged in the market since the introduction of a new type of liquid-based electronic cigarette (e-cigarette) in 2003, which vaporizes a liquid containing nicotine and other substances [8,9]. Philip Morris International’s heat-not-burn (HnB) tobacco, approved by the U.S. Food and Drug Administration as a modified-risk tobacco product, represents one of the recent innovations in tobacco products. HnB, which provides controlled nicotine delivery via heat instead of combustion, is marketed as a potentially safer alternative to traditional smoking [10]. Such HnB tobacco products align with global trends and have gained considerable popularity, including in South Korea, where users perceive them as a relatively less harmful option to smoking [11].

Cigarette smoke (CS), a leading cause of COPD, drives lung damage through p16-mediated senescence [8], mitochondrial dysfunction [12,13], and oxidative stress [9]. Restoring mitochondrial function with alternative oxidase (AOX) reduces CS-induced tissue damage, ROS production, and apoptosis, suggesting the potential to mitigate COPD progression [14]. Previous vapor studies reveal that nicotine-containing e-cigarette vapor extract induces significant inflammation, endothelial damage, and structural changes in the lungs, while nicotine-free e-cigarette vapor extract causes milder effects. Long-term exposure to e-cigarette vapor increases inflammatory cells in the lungs, with more pronounced changes in the nicotine-containing group [15].

However, compared with the extensive research on the harmful effects of traditional tobacco, studies on the effects of emerging tobacco products and nicotine delivery systems such as HnB remain limited. Additionally, despite Philip Morris’s official data claiming lower harmful components in HnB [16], HnB may produce more inflammatory cytokines [17]. Therefore, this study aimed to investigate the harmful effects of these emerging tobacco products and their underlying mechanisms using short-term exposure animal models through proteomic analysis. Furthermore, we comprehensively examined the impact of the new tobacco product compared with the effects of traditional tobacco smoke by comparing the HnB group with a conventional cigarette smoke group, a mixed group of the two, and a control group, all using mice as experimental subjects.

## 2. Results

A total of 18 mice were initially utilized, with each treatment group (the HnB group, tobacco smoking group, and a mixed group exposed to both types of smoke) consisting of five individuals, except for the control group, which comprised three mice. However, during the blood sampling process, one mouse from the cigarette smoke group and one from the HnB group died due to clot formation, resulting in an inability to obtain samples from these two mice.

Despite these challenges, we performed proteomics analysis using a PEA (Olink Target 96 Mouse Exploratory Panel). Cycle threshold (Ct) values were measured for 92 proteins per sample, and normalized protein expression (NPX) values were calculated from Ct values using inter-plate controls. Samples and protein assays were filtered using two strict thresholds. Differences in the expression of the 92 investigated proteins were observed among the three treatment groups and the control group.

### 2.1. Heatmap

**Figure 1:**
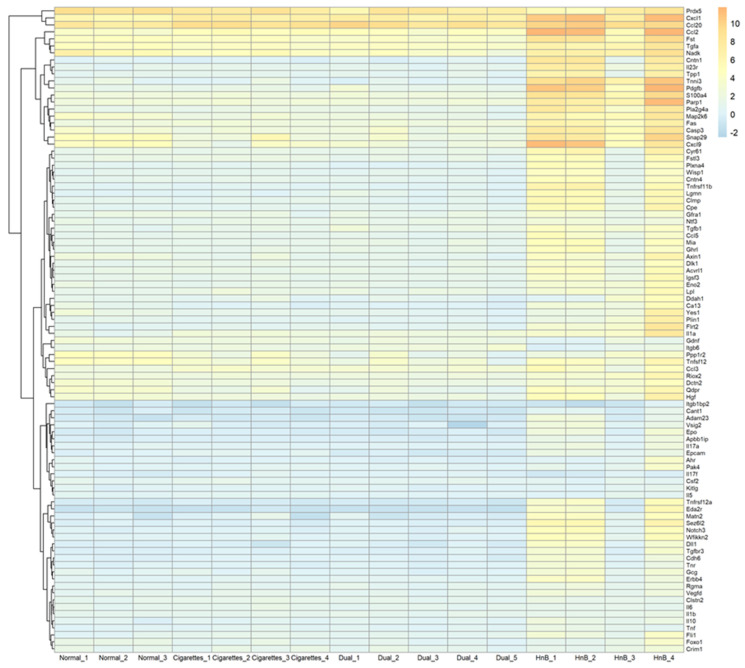
Heatmap illustrating unique proteins. Unique proteins were identified using the proximity extension assay, which was logarithmically scaled, and their normalized protein expression (NPX) values were standardized to zero. Positive values within the heatmap indicate NPX levels that are above the detection threshold and higher than the average scaled NPX value.

Hierarchical clustering analysis with a heatmap (Euclidean distance, complete linkage) clustered the proteins and samples based on their expression, revealing common patterns of grouping among cigarette-exposed mice as identified by the PEA. In the cigarette exposure groups, the levels of various proteins, particularly Ccl20, Cxcl1, and Pdx5, were consistently elevated (Figure 1).

### 2.2. PEA Proteomic Analysis

#### 2.2.1. Comparing Control Mice with Cigarette-Exposed Mice

**Figure 2:**
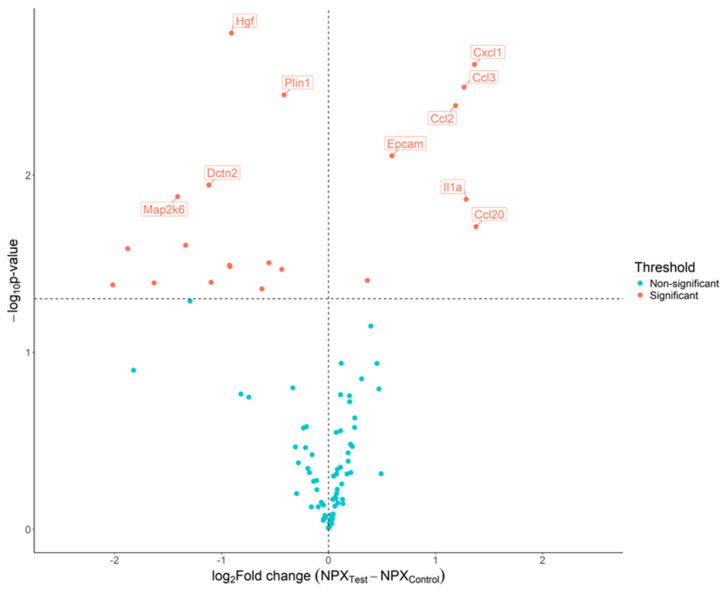
Detection of biomarker candidates using proximity extension assay (PEA) to compare control mice with mice exposed to cigarette smoke. Volcano plot of the 92 proteins analyzed using the PEA. The estimated difference is presented on the x-axis and −log10(*p*-value) is presented on the y-axis. The horizontal dotted line indicates a raw *p*-value of 0.05, and the vertical dotted line indicates the threshold in the log2 ratio of fold change. Dots are colored based on the criteria for significant results.

Comparing the unexposed control mice with those exposed to cigarette smoke revealed notable alterations in the plasma levels of Gdnf, Foxo1, Prdx5, Tgfa, Axin1, Ca13, Ppp1r2, Ccl2, Plin1, Qdpr, Epcam, Ccl3, Hgf, Il1a, Cxcl9, Map2k6, Ccxl1, Dctn2, Tnfsf12, and Ccl20, with increases in Epcam, Ccl2, Ccl3, and Cxcl1, and decreases in Hgf, Plin1, Dctn2, and Map2k6 levels (Figure 2).

#### 2.2.2. Comparing Control Mice with Dual-Smoke-Exposed Mice

**Figure 3:**
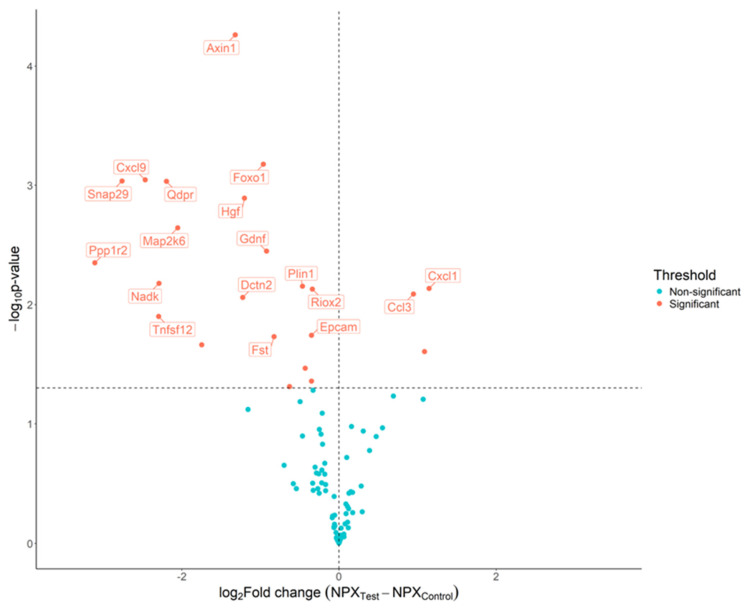
Detection of biomarker candidates using proximity extension assay (PEA) to compare control mice with mice exposed to both cigarette and heat-not-burn smoke. Volcano plot of the 92 proteins analyzed using the PEA. The estimated difference is presented on the x-axis and −log10(*p*-value) is presented on the y-axis. The horizontal dotted line indicates a raw *p*-value of 0.05 and the vertical dotted line indicates the threshold in the log2 ratio of fold change. Dots are colored based on the criteria for significant results.

Comparison of the dual-smoke-exposed mice with the control mice group revealed significant differences in plasma levels of Gdnf, Foxo1, Prdx5, Tgfa, Axin1, Fst, Nadk, Snap29, Ca13, Ppp1r2, Plin1, Qdpr, Riox2, Plxna4, Epcam, Ccl3, Hgf, Il1a, Dadh1, Cxcl9, Map2k6, Ccxl1, Dctn2, and Tnfsf12, with increases in Ccl3 and Cxcl1 and decreases in Axin1, Foxo1, Gdnf, Epo, Nadk, Snap29, Ppp1r2, Qdpr, Hgf, Cxcl9, and Tnfsf12 (Figure 3).

#### 2.2.3. Comparing Control Mice with HnB-Smoke-Exposed Mice

**Figure 4:**
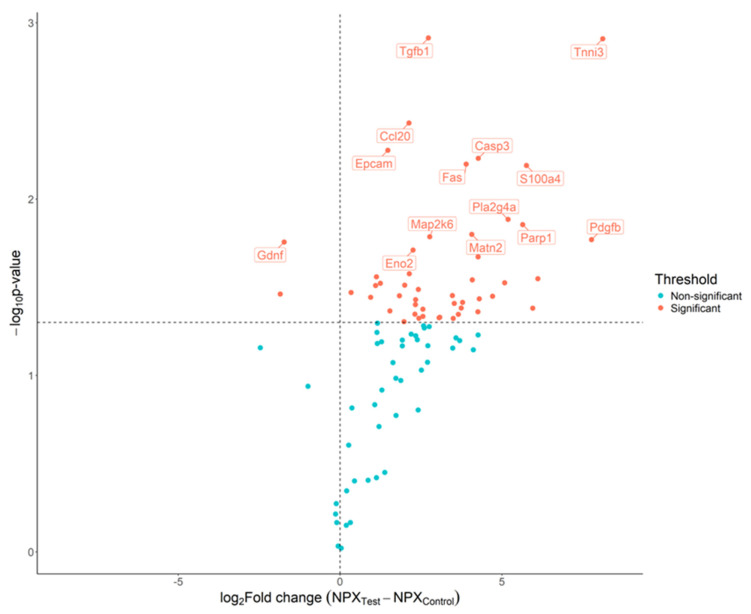
Detection of biomarker candidates using proximity extension assay (PEA) to compare control mice with mice exposed to heat-not-burn smoke. Volcano plot of the 92 proteins analyzed using the proximity extension assay. The estimated difference is presented on the x-axis and −log10(*p*-value) is presented on the y-axis. The horizontal dotted line indicates a raw *p*-value of 0.05 and the vertical dotted line indicates the threshold in the log2 ratio of fold change. Dots are colored based on the criteria for significant results.

Comparing control mice with HnB-smoke-exposed mice revealed significant differences in the plasma levels of Clmp, Matn2, Cpe, Gcg, Gdnf, Tnfrsr11b, Tgfb1, Pla2g4a, Tgfa, Ccl5, Epo, Fst, Rgma, Tnni3, Notch3, Cntn1, S100a4, Ppp1r2, Adam23, Dlk1, Ccl2, Eno2, Wfikkn2, Fas, Plxna4, Epcam, Vsig2, Sez6l2, Il1a, Il23r, Dll1, Il10, Acvrl1, Lgmn, Map2k6, Il1b, Casp3, Apbb1Ip, Wisp1, platelet-derived growth factor (Pdgf) b, Cxcl1, Lpl, Eda2r, Ntf3, Ccl20, Tnr, Parp1, and Tnf, with increases in Tgfb1, S100a4, Fas, Epcam, Plxna4, Casp3, Ccl20, Cxcl9 and Pdgfb levels and decreases in Gdnf and Pplr2 levels (Figure 4).

Comparisons of plasma levels between the control group and the cigarette-exposed, dual-smoke-exposed, and HnB-smoke-exposed mice revealed significant alterations in several proteins. Significant changes, including both upregulation and downregulation of specific markers, are summarized in Table 1. These findings underscore distinct molecular profiles associated with each type of smoke exposure.

## 3. Discussion

In this study, the harmful effects of exposure to smoke from the HnB product were compared with traditional cigarette smoke using an animal model, and the data were analyzed using PEA. Notably, the levels of multiple proteins, including Ccl20, Cxcl1, and Prdx5, increased consistently in the cigarette exposure group, revealing a clear, consistent pattern in the heatmap. Importantly, these increases were also observed in mice exposed to HnB smoke. Additionally, significant elevations in Ccl2, Cxcl9, and Pdgfb levels in HnB-smoke-exposed mice indicate their association with the nicotine pathway; therefore, these characteristic protein profiles may serve as potential biomarkers for understanding the harmful effects of HnB in the future, providing valuable insights for further research and clinical applications.

Both conventional cigarettes and e-cigarettes are primarily designed to deliver nicotine, providing immediate satisfaction through vapors. Traditional cigarettes contain carcinogenic chemicals such as nitrosamines, which are inhaled following combustion, whereas e-cigarettes contain only nicotine and relatively harmless organic solvents. Consequently, e-cigarettes are often portrayed as safer alternatives to tobacco [18]. However, the effects of nicotine on the immune system are nuanced and vary among studies. Notably, cigarette smoke comprises many harmful substances apart from nicotine that can adversely impact health. Nicotine is absorbed into the body via cigarette smoke, triggering various physiological changes, some of which are related to the immune system. Cells of the immune system generate and release various compounds, including cytokines, to regulate inflammation and immune responses in the body. Nicotine can influence the production and release of cytokines, potentially affecting immune responses [19,20]. However, the specific effects may depend on factors such as dose, duration, and frequency of nicotine exposure, as well as individual differences. In this study, we observed elevated levels of Ccl2, Cxcl9, and Pdgfb in mice exposed to e-cigarette smoke, indicating activation of a nicotine-related immune pathway.

CCL2, a chemokine also known as MCP1, plays a crucial role in inflammation and immune responses. It is associated with inflammatory reactions triggered by environmental factors and is released by multiple cells, including macrophages, monocytes, and epithelial cells [21,22]. Notably, CCL2 overexpression influences proliferation, migration, and tumor growth factor (TGF)-β1 expression in lung epithelial cells when exposed to cigarette smoke extract. Additionally, it acts as a regulator in the generation of pro-fibrotic mediators and migration in fibroblasts [23]. Therefore, CCL2 could be vital in controlling extracellular matrix turnover by stimulating intermediary molecules such as TGF-β1, α-smooth muscle actin, and interleukin (IL)-6 in pulmonary fibroblasts. CCL2 may serve as a potential therapeutic target for managing idiopathic pulmonary fibrosis. Other studies suggest that CCL2 is a potent pro-inflammatory chemokine that acts as a chemoattractant for myeloid cells and has been extensively studied as a predictor and potential driver of tumor cell growth and metastasis [24,25].

Smoking tobacco can induce inflammatory and autoimmune diseases via genetic/epigenetic changes, increased oxidative stress, and free radical production, leading to enhanced proliferation of B and T cells, reduced regulatory T cell function, elevated pro-inflammatory cytokines (IL-1β, IL-6, IL-8, and tumor necrosis factors), increased expression of chemotactic cytokines such as recombinant human CXCL9 (MIG), thymus and activation-regulated chemokine, and interferon-inducible T cell α chemoattractant [26,27,28,29,30,31,32,33,34,35,36,37,38]. CXCL9 plays an important role in many diseases, including external infection, autoimmune diseases, tumor treatment, lymphoma [39], and fatty liver disease [40]. Changes in these inflammatory markers and cytokines can lead to cancers at 18 different tumor sites and a range of other chronic diseases, including coronary heart disease, stroke, and chronic obstructive pulmonary disease [41,42]. Notably, research on the specific interaction between CXCL9 and nicotine/e-cigarettes is still evolving, and conclusive evidence remains unavailable. Therefore, it is crucial to consider the broader context of nicotine and e-cigarette use, including their potential effects on overall health and immune function.

The impact of nicotine—present in both tobacco products and e-cigarettes—on various bodily functions has been thoroughly examined. Notably, nicotine exposure may affect the generation and function of growth factors such as PDGFB. PDGFB is a signaling protein implicated in diverse cellular functions, including cell growth, proliferation, and differentiation, and plays a significant role in tissue repair and wound healing. Nonetheless, the precise relationship between PDGFB and nicotine or e-cigarettes is currently under investigation, and conclusive findings have yet to be reached. Von Willebrand factor (vWF), PDGFB, HIVEP1, and GPX3 have been identified as biomarkers associated with venous thromboembolism in the VEBIOS cohort, with the associations of vWF and PDGFB replicated in FARIVE [43]. Notably, various chemicals, such as flavorings and additives in e-cigarettes, may have different effects on the body, and the impact of heating these compounds and their potential toxic effects are understudied. Furthermore, the method of delivering nicotine via e-cigarettes may differ from that of traditional tobacco products, potentially influencing its interaction with signaling proteins such as PDGFB. Current research has indicated elevated PDGFB levels in e-cigarette users; however, the specific effects and mechanisms of these interactions are still being actively studied. As research progresses, a deeper understanding of the relationship between PDGFB and nicotine/e-cigarettes will be obtained.

Although the exact mechanism is unclear, we attempted to identify functional associations between CCL2, CXCL9, and PDGF using the STRING platform (https://string-db.org, accessed on 20 December 2024), as shown in Figure S1. The analysis revealed biological processes associated with cell chemotaxis. Another potential mechanism underlying the observed effects is the increase in levels of damaged mitochondrial DNA in circulating blood, similar to what has been reported for CS [13,44,45]. E-cigarette exposure, like CS, may lead to the release of non-protein pro-inflammatory markers such as cell-free nuclear and mitochondrial DNA [46]. These markers are known to trigger inflammatory responses, suggesting that smoking itself or nicotine exposure could contribute to inflammation through these pathways.

The limitations of this study include the small sample size and the loss of experimental animals during the blood sampling process, which may have affected the robustness of the results. Another limitation is the use of Marlboro Red instead of the standard Coresta 1R6F reference cigarette, which may affect the generalizability of the findings. Finally, a direct comparison between the traditional cigarette group and the HnB group was not undertaken due to the study design, which focused on comparing each group to the control. Nevertheless, this study is strengthened by its comprehensive evaluation of potential biomarkers associated with HnB exposure, providing valuable insights into the effects of alternative nicotine delivery systems on biological pathways and a solid foundation for future research on physiological responses to HnB exposure in humans.

## 4. Materials and Methods

### 4.1. Animal Preparation and Ethical Considerations

Specific pathogen-free male C57BL/6 mice (6 weeks old, weighing 20–25 g) were supplied by RaonBio Inc. (Raonbio, Yongin, Republic of Korea). The mice were housed in a controlled environment at a temperature of 21 ± 2 °C and a relative humidity of 40–60%. They were maintained on a 12-h light/dark cycle and had unrestricted access to food and water. All experimental procedures were approved by the Korea University Institutional Animal Care and Use Committee (Approval Number: KOREA-2022-0066; Approved on: 21 June 2022) and were performed in accordance with the relevant guidelines and regulations. This study complied with the ARRIVE guidelines 2.0 for the transparent and comprehensive reporting of research involving animals.

### 4.2. Grouping and Exposure Protocol

**Figure 5:**
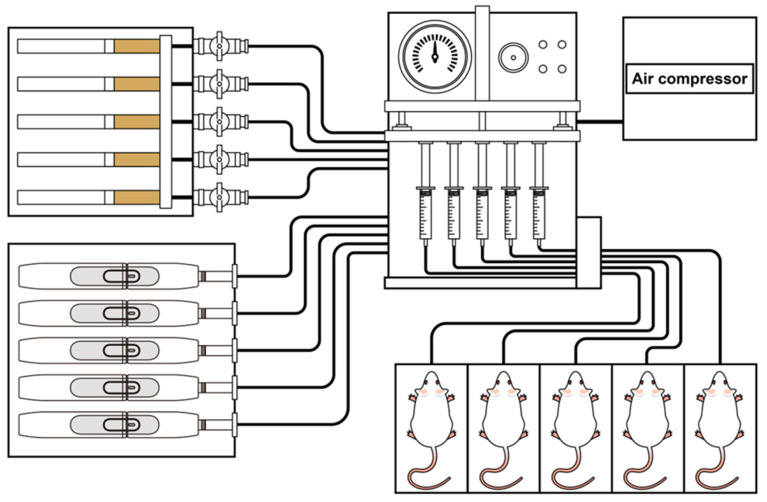
Schematic diagram illustrating smoking exposure to mice. Experimental animals were exposed to tobacco and heat-not-burn smoke five days a week for four weeks using the Smoking Tester Line System of Three Shine Inc. Each group was exposed to the smoke of 20 cigarettes or heat-not-burn sticks, five at a time, over the course of approximately 30–40 min. The mixed-use group was alternately exposed to the smoke of 10 cigarettes and 10 IQOS sticks.

The mice were divided into four groups: each treatment group comprised five mice, while the control group consisted of three mice, resulting in a total of 18 animals. The smaller sample size for the control group was due to ethical considerations in animal experimentation. The treatment groups included the HnB group, the tobacco smoking group, a mixed group exposed to both types of smoke, and a control group exposed only to room air. All mice were placed in a pre-exposure room with air ventilation prior to the exposure. The control group remained in the pre-exposure room and was not subjected to any procedures that could induce stress. In contrast, the exposure groups were placed in a designated smoking room, where they awaited their turn before being exposed to smoking through a smoking machine. The HnB group was exposed to heat-not-burn tobacco, the tobacco smoking group was exposed to conventional cigarette smoke, and the mixed group was exposed to both types of smoke. The experiment utilized the Smoking Tester Line System (Three Shine Inc., Daejeon, Republic of Korea), with each group exposed to tobacco and IQOS smoke 5 days a week for 4 weeks. Each day, the experimental animals were placed in dedicated cages, as shown in Figure 5, and exposed to either 20 cigarettes or IQOS sticks, which were lit in sets of five at a time (taking approximately 30–40 min). The mixed group was alternately exposed to 10 cigarettes and 10 IQOS sticks. In the tobacco smoke group, we used Marlboro Red (Korean Philip Morris, Seoul, Republic of Korea), while the HnB group was exposed to HEETS bronze (Korean Philip Morris, Seoul, Republic of Korea). Following the experiment, the animals were returned to their housing facility after a sufficient period of smoke clearance.

### 4.3. Serum Collection

After the final exposure, the experimental animals were subjected to isoflurane inhalation anesthesia and were operated on by midline incision, exposing the heart and lungs. Subsequently, the animal was euthanized by obtaining blood directly from the heart. During the blood sampling process, one mouse from the cigarette smoke group and one from the HnB group died due to clot formation, which led to an inability to obtain samples from these two mice. The collected blood was centrifuged at 2000× *g* for 10 min and 4 °C, and the supernatant was stored at −70 °C until further analysis.

### 4.4. Proximity Extension Assay (PEA)

Ninety-two proteins in the blood plasma were analyzed using the Olink Multiplex Target 96 Mouse Exploratory Panel (https://olink.com/products/olink-target-96, accessed on 10 March 2024). Each panel was designed with 92 antibody probe pairs that interact with target proteins present in the sample. These panels were selected for their comprehensive coverage of various potential targets associated with critical biological functions, including cell regulation, development, metabolism, and organ damage.

During the reaction, a proximity-dependent DNA polymerization event occurred between a pair of oligonucleotide-labeled antibodies that targeted the protein of interest. This process led to the generation of a PCR reporter sequence, which was subsequently measured using real-time PCR [47,48]. Internal, extension, and detection controls were utilized to monitor deviations, as outlined by the manufacturer (www.olink.com, accessed on 3 May 2024).

Proteins with a call rate below 85%, indicating targets where <85% of individuals exhibited a measurable concentration above the limit of detection, were excluded from further analysis based on the manufacturer’s recommended intra-plate variation. NPX was determined by subtracting an external inter-plate control, with values set relative to a correction factor established by Olink and represented on a log2 scale, with the background level set at 0. Additional details regarding the PEA, including data processing and normalization, can be obtained from the manufacturer’s website (www.olink.com, accessed on 3 May 2024).

### 4.5. Statistical Analysis

Heatmaps were generated using R version 3.5.3 and the “pheatmap” package, involving scaling, normalization, and data reduction. For comparison of multiprotein analyses, NPX was used to denote the protein expression levels. NPX serves as a logarithmic scale for relative quantification, enabling the detection of variations in individual protein levels across samples and facilitating the establishment of protein signatures. NPX values derived from each assay were used for comparative analysis between the three treatment groups and the control. Statistical tests were conducted based on these NPX values, leveraging the group information. The volcano plot displays the estimated difference on the x-axis and −log10(*p*-value) on the y-axis. Horizontal and vertical dotted lines denote a raw *p*-value of 0.05 and a threshold in log2 ratio of fold change, respectively. Dots were color-coded according to criteria for significant results. Box plots were employed to compare protein expression levels across groups, while bar plots were used to visualize fold changes in protein expression for each comparison.

## 5. Conclusions

In conclusion, the increase in the levels of specific proteins, mainly Ccl20, Cxcl1, and Pdx5, observed in both cigarette and HnB users, along with the elevated levels of Ccl2, Cxcl9, and Pdgfr in HnB smokers, indicates an association with the nicotine pathway and identifies potential markers for understanding the harmful effects of HnB. Although nicotine affects the immune system by initiating various bodily changes and altering cytokine release, its precise impact is complex and can be influenced by other toxins in cigarette smoke. Therefore, the immune-modulating effects of nicotine should be analyzed in the broader context of the well-known health risks of smoking. Consequently, further investigation is imperative to fully understand the intricate interplay between nicotine, other cigarette components, and immune function.

## Figures and Tables

**Table 1:** Summary of key plasma protein changes in cigarette-exposed, dual-smoke-exposed, and HnB-smoke-exposed mice compared to controls (Welch’s two-sample *t*-test, *p*-value < 0.05).

	Upregulated	Downregulated
Control vs. Cigarette	Ccl2, Ccl3, Ccxl1, Epcam	Hgf, Plin1
Control vs. Dual smoke exposure	Ccl3, Cxcl1	Axin1. Foxo1, Gdnf, Epo, Nadk, Snap29, Ppp1r2, Qdpr, Hgf, Cxcl9, Tnfsf12
Control vs. HnB smoke exposure	Tnni3, Tgfb1, Ccl2, S100a4, Fas, Epcam, Plxna4, Casp3, Ccl20, Cxcl9, Pdgfb	Gdnf, Ppp1r2

## Data Availability

The datasets generated and/or analyzed during the current study are available from the corresponding author upon reasonable request.

## Supplementary Materials

The following supporting information can be downloaded at https://www.mdpi.com/article/10.3390/ijms26031135/s1.
