# Supplementary material for: Comparative Assessment of Acute Pulmonary Effects Induced by Heat-Not-Burn Tobacco Aerosol Inhalation in a Murine Model
Source: Int J Mol Sci. 2025 Jan 28;26(3):1135. doi: 10.3390/ijms26031135 (PMC11817633; doi:10.3390/ijms26031135)
Supplement: Supplementary file 1 [file ijms-26-01135-s001.zip › ijms-3345366-supplementary.pdf]

Protein assay list

Amyloid beta A4 precursor protein-binding family B member 1-interacting protein (Apbb1ip)
Appetite-regulating hormone (Ghrl)
Aryl hydrocarbon receptor (Ahr)
Axin-1 (Axin1)
C-C motif chemokine 2 (Ccl2)
C-C motif chemokine 20 (Ccl20)
C-C motif chemokine 3 (Ccl3)
C-C motif chemokine 5 (Ccl5)
C-X-C motif chemokine 9 (Cxcl9)
Cadherin-6 (Cdh6)
Calsyntenin-2 (Clstn2)
Carbonic anhydrase 13 (Ca13)
Carboxypeptidase E (Cpe)
Caspase-3 (Casp3)
Contactin-1 (Cntn1)
Contactin-4 (Cntn4)
CXADR-like membrane protein (Clmp)
Cysteine-rich motor neuron 1 protein (Crim1)
Cytosolic phospholipase A2 (Pla2g4a)
Delta-like protein 1 (Dll1)
Dihydropteridine reductase (Qdpr)
Disintegrin and metalloproteinase domain-containing protein 23 (Adam23)
Dual specificity mitogen-activated protein kinase kinase 6 (Map2k6)
Dynactin subunit 2 (Dctn2)
Epithelial cell adhesion molecule (Epcam)
Erythropoietin (Epo)
Follistatin (Fst)
Follistatin-related protein 3 (Fstl3)
Forkhead box protein O1 (Foxo1)
Friend leukemia integration 1 transcription factor (Fli1)
Gamma-enolase (Eno2)
GDNF family receptor alpha-1 (Gfra1)
Glial cell line-derived neurotrophic factor (Gdnf)
Glucagon (Gcg)
Granulocyte-macrophage colony-stimulating factor (Csf2)
Growth-regulated alpha protein (Cxcl1)
Hepatocyte growth factor (Hgf)
Immunoglobulin superfamily member 3 (Igsf3)

Integrin beta-1-binding protein 2 (Itgb1bp2)
Integrin beta-6 (Itgb6)
Interleukin-1 alpha (Il1a)
Interleukin-1 beta (Il1b)
Interleukin-10 (Il10)
Interleukin-17A (Il17a)
Interleukin-17F (Il17f)
Interleukin-23 receptor (Il23r)
Interleukin-5 (Il5)
Interleukin-6 (Il6)
Kit ligand (Kitlg)
Legumain (Lgmn)
Leucine-rich repeat transmembrane protein FLRT2 (Flrt2)
Lipoprotein lipase (Lpl)
Matrilin-2 (Matn2)
Melanoma-derived growth regulatory protein (Mia)
N(G), N(G)-dimethylarginine dimethylaminohydrolase 1 (Ddah1)
NAD kinase (Nadk)
Neurogenic locus notch homolog protein 3 (Notch3)
Neurotrophin-3 (Ntf3)
Perilipin-1 (Plin1)
Peroxiredoxin-5, mitochondrial (Prdx5)
Platelet-derived growth factor subunit B (Pdgfb)
Plexin-A4 (Plxna4)
Poly [ADP-ribose] polymerase 1 (Parp1)
Protein CYR61 (Cyr61)
Protein delta homolog 1 (Dlk1)
Protein phosphatase inhibitor 2 (Ppp1r2)
Protein S100-A4 (S100a4)
Protransforming growth factor alpha (Tgfa)
Receptor tyrosine-protein kinase erbB-4 (Erbb4)
Repulsive guidance molecule A (Rgma)
Ribosomal oxygenase 2 (Riox2)
Seizure 6-like protein 2 (Sez6l2)
Serine/threonine-protein kinase PAK 4 (Pak4)
Serine/threonine-protein kinase receptor R3 (Acvrl1)
Soluble calcium-activated nucleotidase 1 (Cant1)
Synaptosomal-associated protein 29 (Snap29)
Tenascin-R (Tnr)
Transforming growth factor beta receptor type 3 (Tgfbr3)
Latency-associated peptide transforming growth factor beta-1 (Tgfb1)

Tripeptidyl-peptidase 1 (Tpp1)
Troponin I, cardiac muscle (Tnni3)
Tumor necrosis factor (Tnf)
Tumor necrosis factor ligand superfamily member 12 (Tnfsf12)
Tumor necrosis factor receptor superfamily member 11B (Tnfrsf11b)
Tumor necrosis factor receptor superfamily member 12A (Tnfrsf12a)
Tumor necrosis factor receptor superfamily member 27 (Eda2r)
Tumor necrosis factor receptor superfamily member 6 (Fas)
Tyrosine-protein kinase Yes (Yes1)
V-set and immunoglobulin domain-containing protein 2 (Vsig2)
Vascular endothelial growth factor D (Vegfd)
WAP, Kazal, immunoglobulin, Kunitz and NTR domain-containing protein 2 (Wfikkn2)
WNT1-inducible-signaling pathway protein 1 (Wisp1)

Supplementary Figure

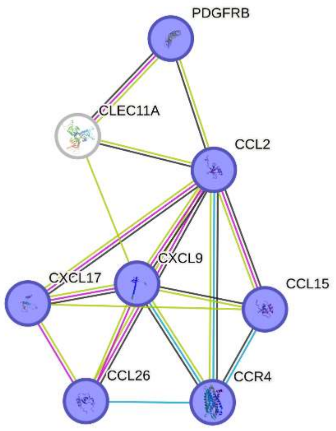

Figure S1. STRING analysis of functional associations between CCL2, CXCL9, and PDGF. The network highlights biological processes linked to cell chemotaxis.
